# Insights into the genetic variability and evolutionary dynamics of tomato spotted wilt orthotospovirus in China

**DOI:** 10.1186/s12864-023-09951-9

**Published:** 2024-01-08

**Authors:** Ali Kamran, Ying Li, Wanhong Zhang, Yubin Jiao, Tahir Farooq, Yong Wang, Dongyang Liu, Lianqiang Jiang, Lili Shen, Fenglong Wang, Jinguang Yang

**Affiliations:** 1 Key Laboratory of Tobacco Pest Monitoring, Controlling & Integrated Management, Tobacco Research Institute of Chinese Academy of Agricultural Sciences, 266101 Qingdao, China; 2 Graduate School of Chinese Academy of Agricultural Sciences, 100081 Beijing, China; 3 Key Laboratory of Agricultural Microbiology, College of Agriculture, Guizhou University, 550025 Guiyang, China; 4 Guangdong Provincial Key Laboratory of High Technology for Plant Protection, Plant Protection Research Institute, Guangdong Academy of Agricultural Sciences, 510640 Guangzhou, China; 5 Tobacco Company of Yunnan Province, Liangshan Company, 615000 Xichang, Sichuan China

**Keywords:** Tomato spotted wilt orthotospovirus, Phylogenetic analysis, Recombination, Reassortment, Genetic diversity, Population dynamics, statistical estimation

## Abstract

**Background:**

Viral diseases are posing threat to annual production and quality of tobacco in China. Recently, tomato spotted wilt orthotospovirus (TSWV) has been reported to infect three major crops including tobacco. Current study was aimed to investigate the population dynamics and molecular diversity of the TSWV. In the current study, to assess and identify the prevalence and evolutionary history of TSWV in tobacco crops in China, full-length genome sequences of TSWV isolates from tobacco, were identified and analyzed.

**Methods:**

After trimming and validation, sequences of new isolates were submitted to GenBank. We identified the full-length genomes of ten TSWV isolates, infecting tobacco plants from various regions of China. Besides these, six isolates were partially sequenced. Phylogenetic analysis was performed to assess the relativeness of newly identified sequences and corresponding sequences from GenBank. Recombination and population dynamics analysis was performed using RDP4, RAT, and statistical estimation. Reassortment analysis was performed using MegaX software.

**Results:**

Phylogenetic analysis of 41 newly identified sequences, depicted that the majority of the Chinese isolates have separate placement in the tree. RDP4 software predicted that RNA M of newly reported isolate YNKM-2 had a recombinant region spanning from 3111 to 3811 bp. The indication of parental sequences (YNKMXD and YNHHKY) from newly identified isolates, revealed the conservation of local TSWV population. Genetic diversity and population dynamics analysis also support the same trend. RNA M was highlighted to be more capable of mutating or evolving as revealed by data obtained from RDP4, RAT, population dynamics, and phylogenetic analyses. Reassortment analysis revealed that it might have happened in L segment of TSWV isolate YNKMXD (reported herein).

**Conclusion:**

Taken together, this is the first detailed study revealing the pattern of TWSV genetic diversity, and population dynamics helping to better understand the ability of this pathogen to drastically reduce the tobacco production in China. Also, this is a valuable addition to the existing worldwide profile of TSWV, especially in China, where a few studies related to TSWV have been reported including only one complete genome of this virus isolated from tobacco plants.

**Supplementary Information:**

The online version contains supplementary material available at 10.1186/s12864-023-09951-9.

## Background

Tobacco is an important cash crop around the globe, despite its gradually reducing production owing to different factors. Whereas, China is a world-leading producer of tobacco (*Nicotiana tabacum*) where viral diseases are posing threat to annual production and quality of the final product. The tobacco industry in China remains one of the most important sources of tax revenue for the central government [1]. According to Statistics Division of Food and Agriculture Organization of the United Nations (FAOSTAT), 2020, China was the world-leading tobacco producer with 9,39,054 ha of cultivated area, producing 22,738 hg/ha annually (http://www.fao.org/faostat/en/#data/QC). Tomato spotted wilt orthotospovirus (TSWV) is one of the most devastating plant viruses, infecting more than 1000 plant species and causes significant economic losses to many agronomic and horticultural crops [2, 3]. The global spread of TSWV can be attributed to its efficient insect vector, western flower thrips (*Frankliniella occidentalis*) which is highly fecund, needs a short generation time, is highly locomotive, prefers concealed spaces, and has polyphagous nature. Additionally, it has developed resistance against several insecticides that further increased the spread of its vectored plant viruses including TSWV [4]. With the elevated volume of international trade in agricultural products; *orthotospoviruses*, especially TSWV and its vector have spread across Asian countries including China, infecting vegetables and horticultural crops. To date, nine members of *orthotospovirus* have been reported to infect economically important crops in Yunnan, Guizhou, and Guangxi provinces in southwest China [5]. While, TSWV is consistently reporting to infect more valuable plant species in China [6, 7].

TSWV, an enveloped, negative-strand RNA virus is the type member of the genus *orthotospovirus* i.e., the only plant-infecting genus within the family *tospoviridae*. TSWV is considered one of the ten most devastating plant viruses due to the ubiquitous nature of the thrips vector and the extremely wide host range of the virus [8]. The losses associated with TSWV and other tospoviruses have exceeded tens of millions of dollars worldwide [8–10].

TSWV genome consists of three RNA segments, denoted as large (L), medium (M), and small (S) that code for the viral RNA-dependent RNA polymerase (RdRp), cell-to-cell movement protein (NSm), the glycoproteins precursor (GP), a nonstructural protein (NSs), and the nucleocapsid protein (N), respectively [11–13]. L RNA is negative-sense, the M and S genomic RNAs have ambisense genomic organization. The GP precursor is changed by the host proteases into two viral glycoproteins Gc (78 kDa), and Gn (58 kDa). Virions are spherical having a diameter of ∼80–120 nm and covered with a phospholipid membrane. Spikes on the outer surface are the result of embedded Gc and Gn in the lipid membrane. The core region that makes the minimal infectious unit of mature virus particles, houses all three genomic RNAs firmly packaged with the N protein and a little amount of the RdRp (of viral origin) into ribonucleic (capsid) proteins (RNPs) [13–15]. Due to the identification of an increasing number of new viruses, and the building up of sophisticated knowledge about this versatile plant virus group, the previously designated family *bunyaviridae*, and genus *tospoviruses* are now classified as a separate family *Tospoviridae* and genus *orthotospovirus* [16]. There are three main stages during the establishment of successful infection and dissemination of TSWV: (a) replication and transcription of the genetic material to harvest huge numbers of infectious RNPs; (b) subsequent inter/intracellular trafficking of these RNPs; and (c) enveloping by Golgi apparatus membranes to help virus acquisition by insect vectors, resulting in the spread of TSWV to uninfected crop plants. To successfully maintain these stages, all the *orthotospoviruses* have evolved to manipulate the host cellular machinery smartly, but it is mainly during the very first stage that RNAi and host defense based on *R* gene are produced [17].

Despite the placement among the ten most devastating plant viruses, only a few full-length genomes of TSWV have been studied worldwide, although having an increasing trend, including but not restricted to Australia, China, South Korea, Spain, and the USA [5, 18–22]. In the current study, to assess and identify the prevalence and evolutionary history of TSWV in tobacco crops in China, full-length genome sequences of ten TSWV isolates from tobacco, were identified and analyzed. Besides these full-length genomes, six partial genomes were also annotated whose complete RNA segments were sequenced but all three segments could not be retrieved. This is the first time that several isolates of TSWV are retrieved from a single host i.e., tobacco. To achieve this goal, a large landscape of important tobacco-growing areas was surveyed including Hunan, Shandong, Shanxi, Sichuan, and Yunnan provinces. Molecular phylogenetic analysis depicted that all the Chinese isolates of TSWV have separate placements from previously reported isolates around the globe depicting the conservation of the Chinese population. This trend was also pertained by RDP4, RAT, and population dynamics analysis. Taken together, this is a valuable addition to the existing worldwide profile of TSWV, especially in China, where a few studies related to TSWV have been reported including only one complete genome of this virus isolated from tobacco plants.

## Results

### PCR assay for TSWV full genome analysis

**Fig. 1:**
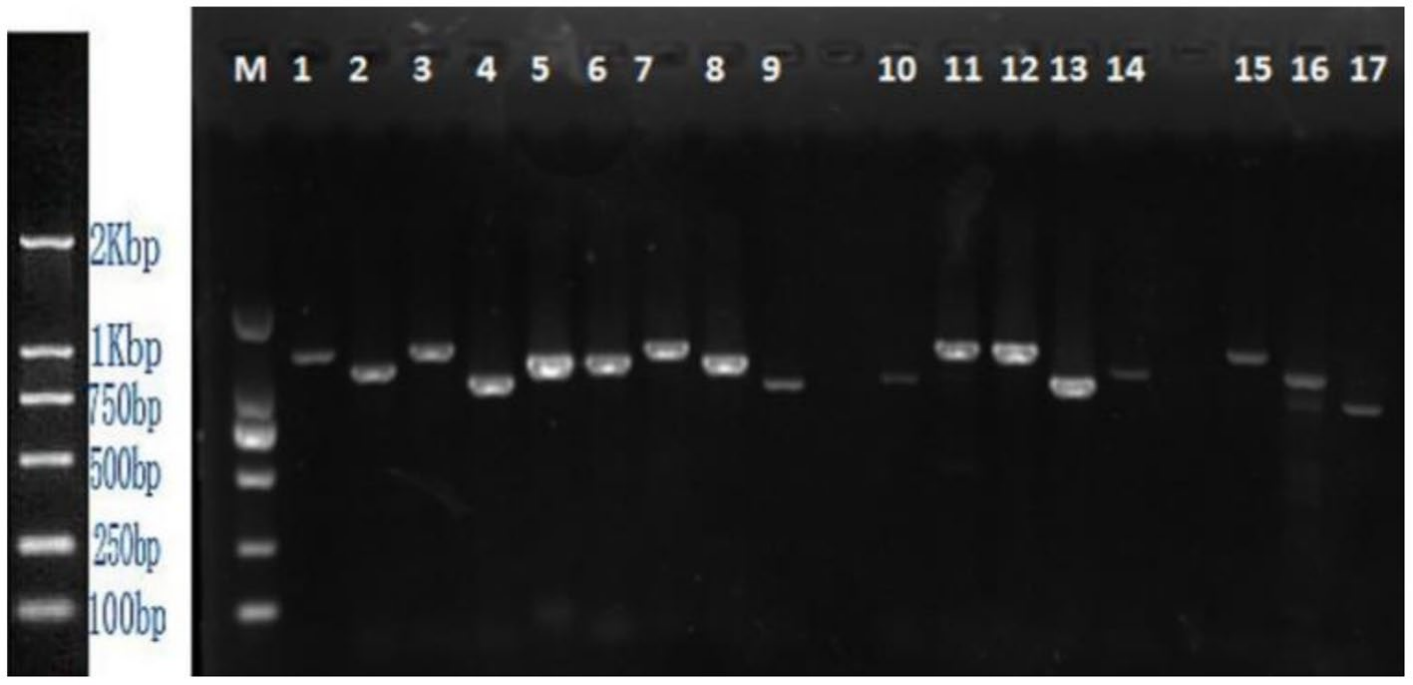
Representative 1.5% agarose gel showing expected amplicons of all three genomic RNAs of YNHH isolate of TSWV: left to right; Lane M: DL2000 ladder; Lane 1–9: RNA-L; Lane 10–14: RNA-M; Lane 15–17: RNA-S

Total RNA extracted from 156 leaf samples (83% were positive for TSWV infection) was converted to cDNA through RT-PCR assay, using different primer pairs constructed to target each genomic segment. The expected amplicons were retrieved from 80% of tested positive samples collected from Hunan, Shaanxi, Shandong, Sichuan and Yunnan provinces (Fig. 1). Original photo of 1.5% agarose gel was also provided (Suppl. figure S1).The incidence of viral infection was highest in Yunnan (85%), and lowest in Sichuan (35%). While, TSWV was not detected in any of the tested samples collected from Gansu province.

### Full genome sequence analysis and molecular phylogeny test

A total of 41 full-length sequences were retrieved for all three genomic segments i.e., L (15), M (16), and S (10) of TSWV from the tobacco plant. From these sequences, ten isolates were comprised of full-length genomes, including all three segments (YNHH; MN833244, YNKM-1; MN865481, YNQJ; MN833245, SDLY-1; MN833242, YNHHMZ; MN870626, YNKMSL; MN833246, YNHHML; MN833247, YNHHXD; MN833249, YNHHLX; MN833250, and YNHHSP; MN833252). Whereas, six isolates were partially sequenced. All the accession numbers of GenBank submitted sequences are listed in Table 1. Amino acid similarity analysis showed that L segment had a similarity between 100 − 94.2%, where YNHHKY, and YNHHMZ (new sequences) were 100% similar whereas MK682812 (capsicum, China), and JF960237 (tomato, China) had lowest similarity (94.2%) among tested sequences. M segment showed aa identity between 99.93 and 85.41%, where YNHHKY, and YNHHMZ had the highest and MG602672 (chrysanthemum, Zimbabwe) and KM657117 (red pepper, China) showed the lowest similarity percentage. Moreover, S segment depicted 99.45–88.35% similarity, where KM657114 (red pepper, China) and KM657115 (tobacco, China) had highest similarity and MG989676 (pepper, Italy) and KT160282 (pepper-USA) had the minimum identity percentage (Suppl. table S1- S3). The lower number of retrieved S segments may attribute towards the smaller size and extremely low titer that hindered the amplification from other samples.

**Table 1:** List of GenBank accession numbers of complete genome sequences corresponding to three segments of TSWV isolates identified in the current study

Number	Collection site	Isolate name	L^a^	M^a^	S^a^
1	Shanxi, shangluo	SXSL	---	MN870627	---
2	Sichuan, liangshan	SCLS	MN833243	MN833253	---
3	Yunnan, honghe	YNHH	MN833244	ON496935	MN861975
4	Yunnan, kunming	YNKM-1	MN865481	MN870628	MN861976
5	Yunnan, qujing	YNQJ	MN833245	MN870639	MN861977
6	Shandong, linyi	SDLY-1	MN833242	MN870629	MN861978
7	Yunnan, honghe	YNHHMZ	MN870626	MN870630	MN861979
8	Yunnan, kunming	YNKMSL	MN833246	MN870631	MN861980
9	Yunnan, honghe	YNHHML	MN833247	MN870632	MN861981
10	Yunnan, honghe	YNHHGJ	MN833248	MN870633	---
11	Yunnan, kunming	YNHHXD	MN833249	MN870634	MN861982
12	Yunnan, honghe	YNHHLX	MN833250	MN870635	MN861983
13	Yunnan, kunming	YNKM-2	MN870625	MN870636	---
14	Yunnan, honghe	YNHHKY	MN833251	MN870637	---
15	Yunnan, honghe	YNHHSP	MN833252	MN870638	MN861984
16	Hunan changsha	HNCS	MN861974	MN870640	---

^a^ Genomic segment: L RNA, M RNA, and S RNA of TSWV genome

**Fig. 2:**
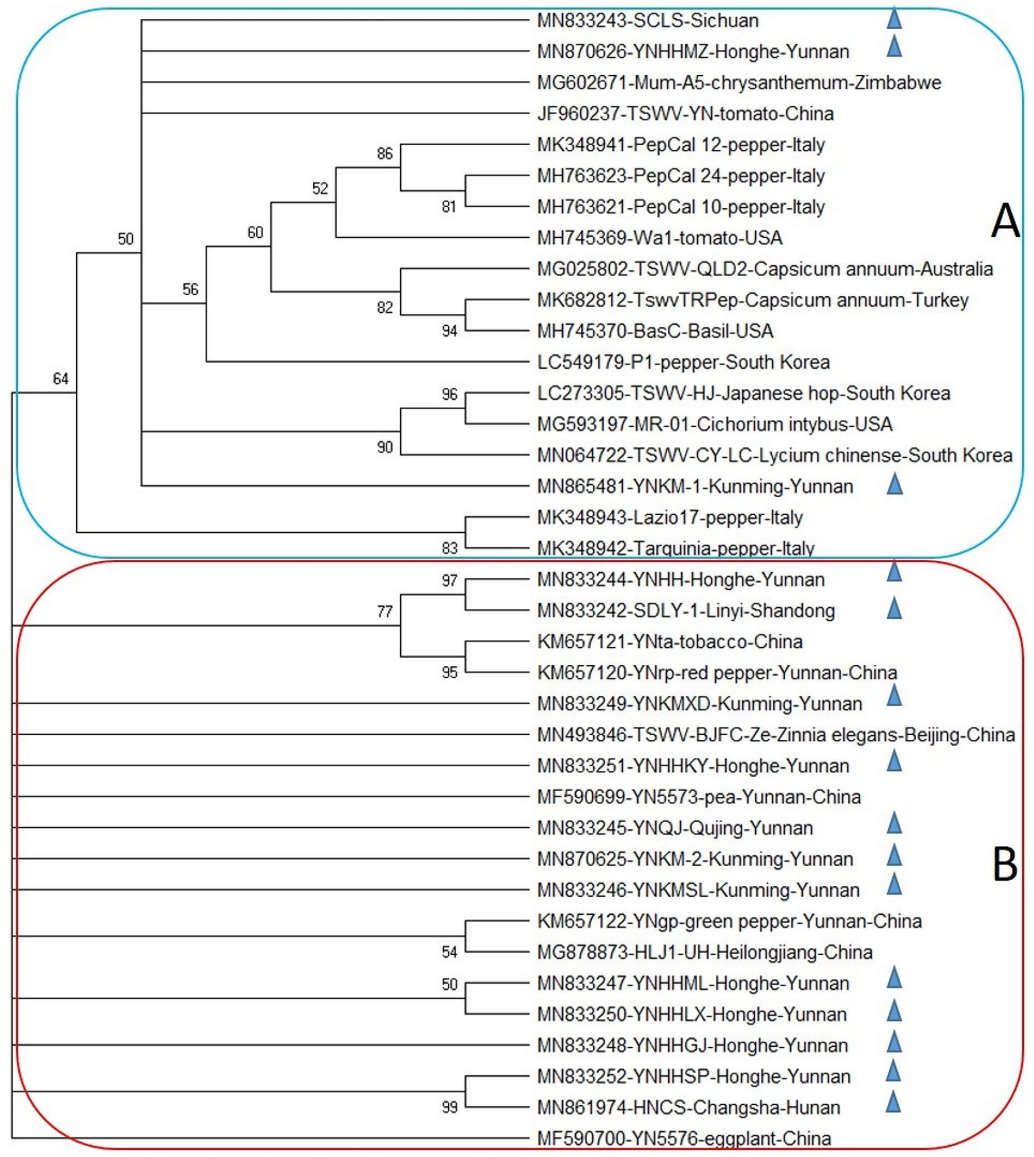
Phylogenetic tree based on the complete nucleotide sequences of genomic segment L of previously reported TSWV isolates and fifteen segments sequenced herein from tobacco plant (denoted by symbol▲). The bootstrap consensus tree was constructed using the Maximum likelihood (ML) method in the MegaX program. Bootstrap analysis was set at 1000 replicates

A phylogeny test was performed among all three genomic RNA segments of newly sequenced TSWV Chinese isolates and the corresponding sequences retrieved from GenBank. All the fifteen new RNA L segments of TSWV were phylogenetically analyzed along with 22 previously reported corresponding sequences. The maximum likelihood tree in MegaX software revealed two distinct clades A and B. Where clade A mostly contained worldwide sequences from Australia, Italy, South Korea, Turkey, USA, and Zimbabwe, except three newly reported RNAs i.e., one from Sichuan province (SCLS) and the other two from Yunnan province (YNHHMZ and YNKM-1), and one previously reported isolate TSWV-YN from China. Whereas, the rest of the twelve new L RNAs from Hunan, Shandong, and Yunnan tended to be closely related to Chinese sequences from different host plants including MF590700 (eggplant; Yunnan), KM657122, (green pepper; Kunming), MG878873 (Heilongjiang), MF590699 (Pea, Yunnan), JF960237 (tomato; Yunnan), MN493846 (*Zinnia elegans*; Beijing), KM657121 (tobacco, Yunnan), and KM657120 (red pepper, Yunnan) together in clade B. (Fig. 2).

**Fig. 3:**
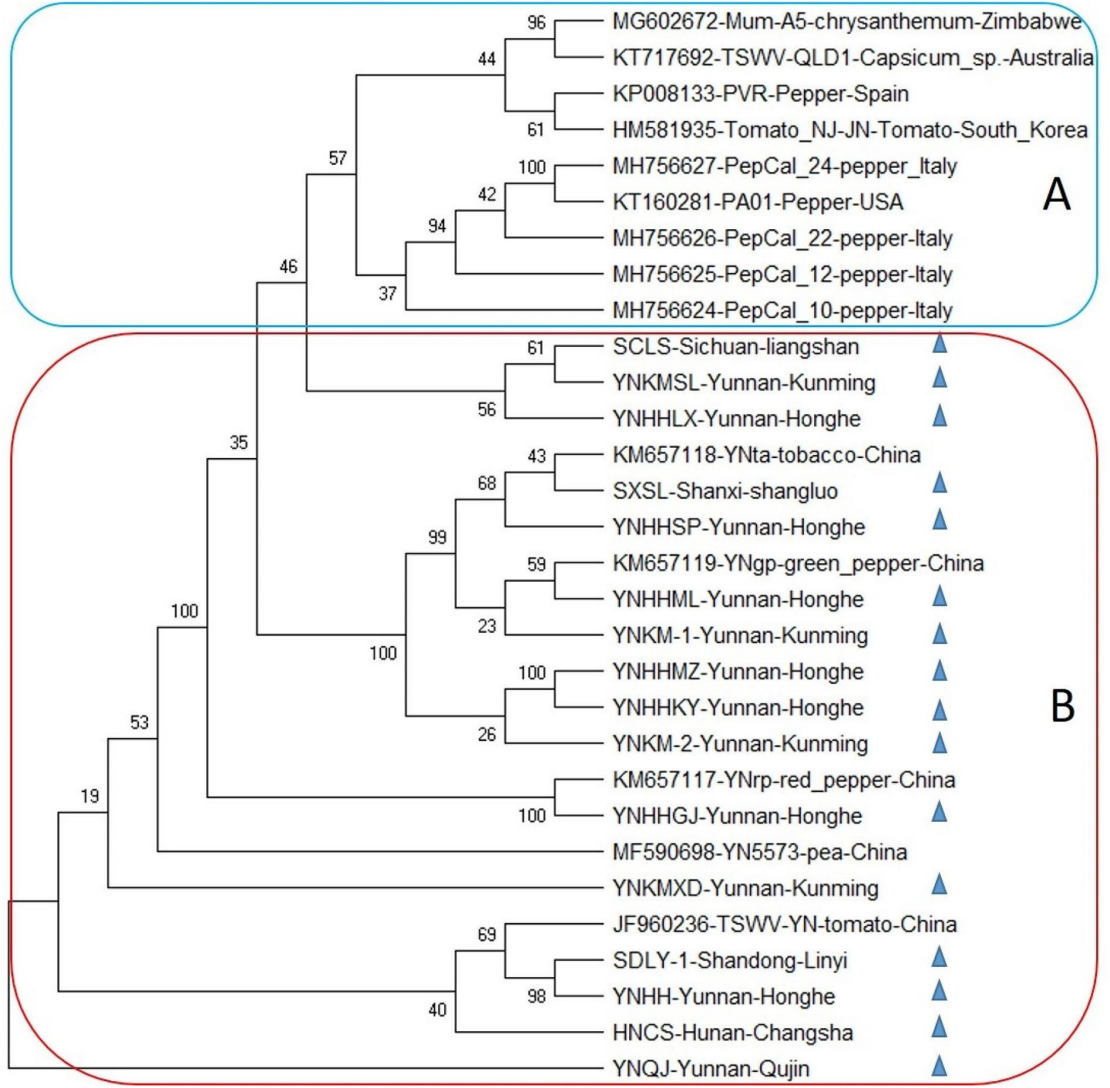
Phylogenetic tree based on the complete nucleotide sequences of genomic segment M of previously reported TSWV isolates and sixteen segments sequenced herein from tobacco plant (denoted by symbol▲). The bootstrap consensus tree was constructed using the Maximum likelihood (ML) method in MegaX program. Bootstrap analysis was set at 1000 replicates

**Fig. 4:**
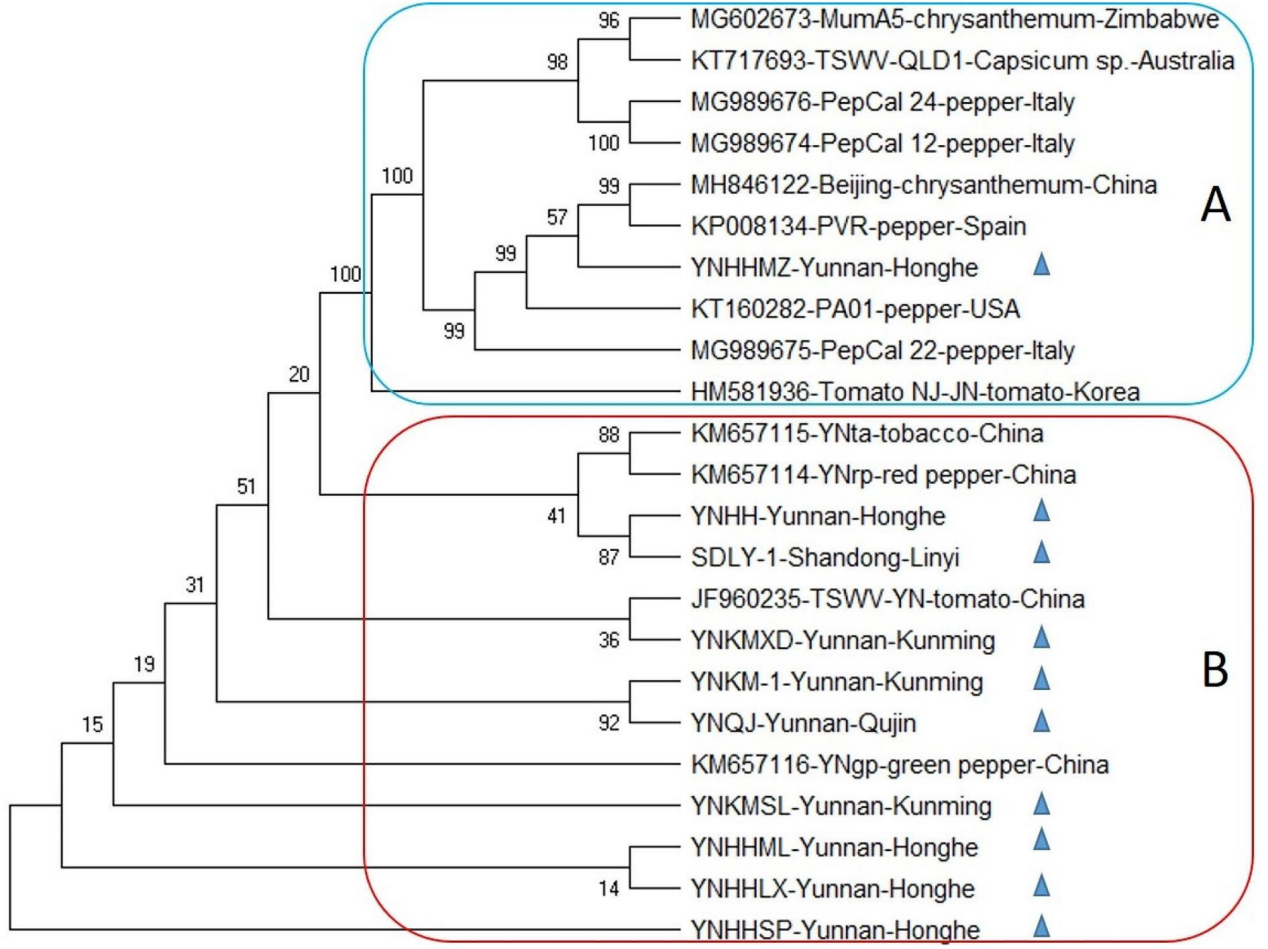
Phylogenetic tree based on the complete nucleotide sequences of genomic segment S of previously reported TSWV isolates and ten segments sequenced herein from tobacco plant (denoted by symbol▲). The tree was constructed using the Maximum likelihood (ML) method using the MEGAX program. Bootstrap analysis was set at 1000 replicates.All the inoculated tobacco plants showed symptoms ten days post-inoculation (dpi). The plants inoculated with inoculum sources from Changsha showed severe symptoms compared to the ones inoculated with inoculum sources from Kunming. TSWV infection was re-confirmed through RT-PCR in all the inoculated plants. The positively detected plants were maintained as the virus source in the greenhouse. Tomato plants were then inoculated using the inoculum source multiplied in tobacco plants in the greenhouse to preserve the TSWV source in the primary host plant for long-term usage. As the tobacco plants died after a few days of showing TSWV symptoms (personal observation). While all the inoculated tomato plants showed TSWV symptoms. All the tested samples showed amplicons of the expected size in 1% agarose gel

The bootstrap consensus phylogenetic tree of newly reported RNA M of TSWV isolates divided mainly into two formations A and B (Fig. 4). Where formation A contains ten isolates from Zimbabwe, Australia, Italy, Spain, South Korea, and the USA, infecting Chrysanthemum, pepper, *Capsicum* sp., and tomato plants. Whereas, all the Chinese isolates were placed in formation B, comprising five previously reported isolates from tobacco, tomato, pea and pepper plants, and sixteen newly reported segments, infecting tobacco plants from Shanxi, Shandong, Sichuan, and Yunnan provinces. Newly reported RNA M isolate YNQJ from Yunnan made separate root in the tree showing the distant relationship with other isolates (Fig. 3).

Following the same pattern as RNA L and RNA M, the phylogenetic tree of RNA S also showed the trend of separation of Chinese isolates from the rest of the world sequences except for one newly reported YNHHMZ isolate that has a close relationship with Spanish isolate PVR. In detail, all 23 RNA S isolates including ten new sequences divided into two main clades i.e., global (A) and Chinese (B). Where clade A of the tree housed all the sequences from Zimbabwe, Australia, Italy, Spain, South Korea, USA, along with one Chinese isolate mentioned above. From an evolutionary point of view, it was interesting that this isolate might share a close origin with the Spanish population of TSWV, which indicates the versatile ability of the virus to modify genomic segments for evolution. The tree was rooted in YNHHSP isolate from Yunnan province (Fig. 4).

### Recombination analysis

**Fig. 5:**
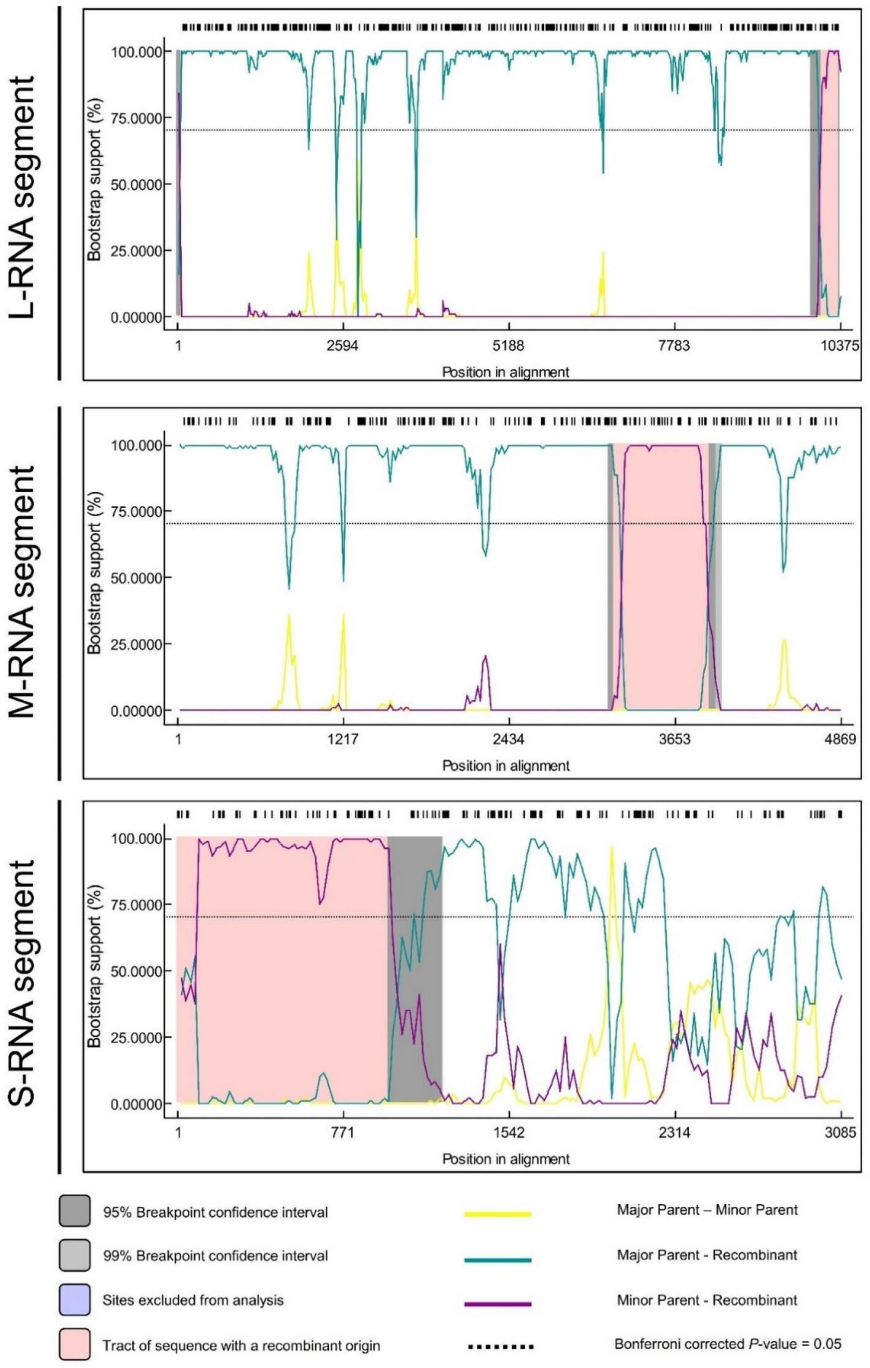
Graphical representation of potential recombination breakpoints detected among TSWV genomic segments: L, M, and S RNAs. Each colored line represents major or minor recombinant isolates and the area showing recombination is represented by pink color

To investigate the genetic diversity, we performed recombination analysis through RDP4 equipped with different detection algorithms. For all the analyzed sequences, only the distinct recombination events confirmed by at least three detection algorithms supported by a P-value of < 0.001 were set as reliable (Table 2; Fig. 5). The results showed that for 37 L segments of TSWV, one and two recombination events were detected in MK348942 from Italy (*P*-value = 2.058 × 10^− 16^) and KP008132 from Spain (*P*-value = 2.279 × 10^− 09^). Although, the potential recombination breakpoint on the genomic region was not too long i.e., ranging from 8665 to 8886 bp (without gaps) for the Italian isolate, and 8838 to 8913 bp (without gaps) for the Spanish one. Events were predicted by GeneConv, Bootscan and 3SEQ in both sequences. While the major and minor parents were detected as MK348943 and MH763623 for Italian and LC549179 and MK682812 for Spanish L isolate, respectively (Table 2).

**Table 2:** Recombination events with high significance as detected by RDP in L, M and S segments of TSWV

Recombination event	Sequences detected with recomb. event	Recombinant sequence^1^		Recombination breakpoints without (with) gaps		Parental sequences		Detection Methods^2^	*P*-value^3^
		Isolate	Country	Begin	End	Major	Minor		
**L-RNA segment**									
1	1	MK348942	Italy	8665 (10,084)	8886 (10,348)	MK348943	MH763623	**G**B3	2.058 × 10^− 16^
2	1	KP008132	Spain	8838 (10,284)	8913 (10,374)	LC549179	MK682812	**G**B3	2.279 × 10^− 09^
**M-RNA segment**									
1	1	YNKM-2	China	3111 (3208)	3811 (3908)	YNKMXD	YNHHKY	RG**B**MCS3	6.586 × 10^− 18^
**S-RNA segment**									
1	1	MG989675	Italy	0 (1)	977 (980)	MH846122	MG989676	RG**B**MCS3	1.395 × 10^− 07^
2	2	MG989674	Italy	879 (882)	1973 (1880)	MG602673	HM581936	**C**S3	4.933 × 10^− 03^

^1^ R, RDP; G, GeneConv; B, Bootscan; M, MaxChi; C, CHIMAERA; S, SisScan; 3, 3SEQ; ^2,^^3^ The described P-value corresponds to the calculated P-value for the event in question, detected by the program in bold and underlined

**Fig. 6:**
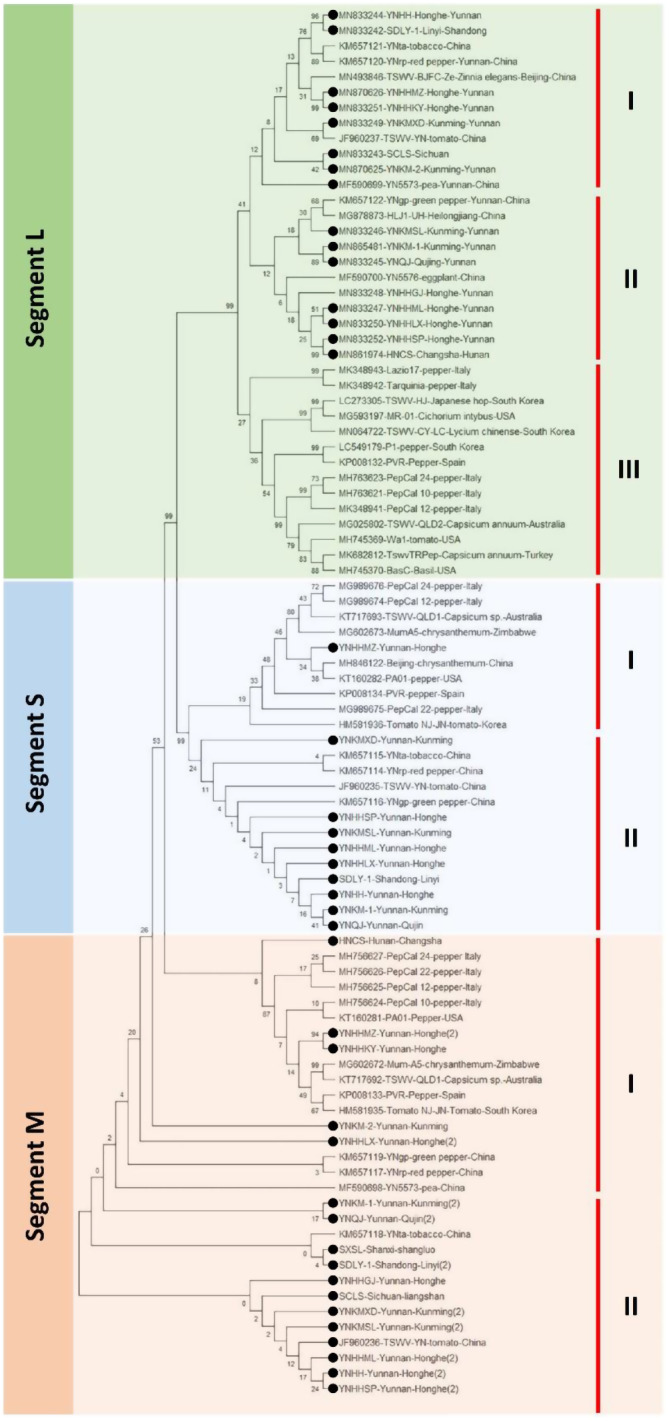
The reassortment neighbor-joining (NJ) tree comprising complete genomes of TSWV reported herein and corresponding GenBank sequences: sequences denoted with symbol ⚫ are reported in current study. The tree was constructed in MAGAX software with bootstrap value set at 1000 replicates

Whereas, RNA M of newly reported Chinese isolate YNKM-2 had one highly significant recombination event (*P*-value = 6.586 × 10^− 18^) spanning from 3111 to 3811 bp (without gaps), detected by seven algorithms i.e., RDP, GeneConv, Bootscan, MaxChi, CHIMAERA, SisScan, and 3SEQ. For the only RNA M recombinant, YNKMXD and YNHHKY were detected as major and minor parents, respectively (Table 2; Fig. 5). Furthermore, it was interesting to have around 2 kb recombinant genomic area in the case of RNA S genomic segment, spanning collectively from 01 to 1973 bp comprising of three recombinant sequences; MG989675, and MG989674 (both from Italy) with one and two recombination events, respectively. Isolate MG989675 with a recombinant area of 01 to 977 bp (*P*-value = 1.395 × 10^− 07^) had major and minor parents as MH846122, and MG989676, respectively. The event was detected by seven algorithms applied as default. Whereas, MG989674 isolate having recombinant region from 879 to 1973 bp (without gaps) (*P*-value = 4.933 × 10^− 03^), tended to have major and minor parental sequences as MG602673, and HM581936, respectively (Table 2; Fig. 5). Additionally, the breakpoint area with 95% confidence interval of RNA S was much larger than that of segment L and M. RAT analysis for the possible recombinant sequences showed the same trend as compared to RDP4 results (Suppl. figure S1-3). Additionally, to assess the reassortment events amongst newly identified three segments i.e. L, M, and S; the sequences were amalgamate and a separate analysis was performed using RDP4. The results showed only one relatively shorter region of possible reassortment in case of M segment of YNKMXD and YNHHKY. The same trend was also observed in above mentioned RDP4 analysis. Whereas, L and S segments of new isolates did not depict any reassortment event (Suppl. figure S5). To further clarify the reassortment events among all the selected isolates of TWSV from GenBank and reported herein, a comprehensive genome reassortment analysis was conducted performing phylogeny test [23] of complete genome sequences of TSWV through MAGAX software. Neighbor-joining (NJ) tree depicted that L segment of YNKMXD isolate has close relationship with previously reported tomato-infecting Chinese isolate. While the S segment of same isolate shared root with tobacco and red-pepper isolates, reported from China. More interestingly, S segment of YNHHMZ isolate shared root with chrysanthemum-infecting isolate from China and pepper-infecting isolate from USA. On the other hand, M segment of isolate HNSC occupied position close to European rather that Chinese isolates. While the M segment of YNHHMZ and YNHHKY isolates has clade-sharing with Zimbabwean, Australian, Spanish and Korean segments (Fig. 6).

### Genetic diversity and population dynamics of both Chinese and global TSWV isolates

#### Estimation of genetic diversity

The quantitative estimation of the genetic diversity of three genomic segments L, M, and S of TSWV revealed the trend of low genetic variation computing the π (< 0.1) among all the current global TSWV segments (Table 3). Among Chinese isolates it was considerably low (0.004 to 0.014), indicating a very low genetic diversity of this virus in this region. The L segment (Chinese origin) was shown to be most conserved having the lowest π value (0.004). However, it was interesting to note that the total number of mutations (Eta) was very low for the Chinese L segment as compared to the global (364 and 1402, respectively), which is an indication of the stability of RdRp structure of Chinese isolates, even though the analyzed sequences were 23 and 14, respectively. However, the number of distinct haplotypes (h) was higher in the case of L segment of the Chinese population (21) in comparison to the global (14), but the haplotype diversity (hd) remained almost consistent between both populations (0.992 to 1.000). The number of polymorphic sites (S) depicted the same trend of less intra-divergence of Chinese isolates, in contrast to global segments where the value of S was relatively higher. S RNA of both Chinese and global origin showed less divergence i.e., 224 from 15 sequences, and 306 from eight sequences, respectively. While M segment of the Chinese population depicted the highest S value of 372 from 21 sequences, followed by L segment having 361 segregating sites from 23 sequences (Table 3).

**Table 3:** Estimation of genetic diversity of the complete genomic segments; L, S, and M RNA of TSWV

TSWV Population	TSWV segment	No. of sequences	No. of analyzed sites	S	h	Hd	*π*	*θw*		Eta
								Per site	Per sequence	
**China**	L	23	8903	361	21	0.992	0.004	0.011	98.623	364
	S	15	2905	224	15	1.000	0.014	0.024	70.120	228
	M	21	4760	372	21	1.000	0.012	0.021	104.510	376
**Global**	L	14	10,370	1336	14	1.000	0.039	0.047	422.466	1402
	S	8	2835	306	8	1.000	0.038	0.042	121.487	315
	M	9	4755	613	9	1.000	0.042	0.048	232.168	631

S: number of polymorphic (segregating) sites, h: number of haplotypes, Hd: haplotype (gene) diversity, π: nucleotide diversity (per site), Eta: total number of mutations, *θw*: Watterson’s theta

#### Statistical evaluation of demographic changes through neutrality tests

The neutral evolution test was analyzed on the bases of the total number of mutations (Eta) and segregating sites (S). All three tests (Tajima’s D, Fu and Li’s D and Fu and Li’s F) revealed statistically significant and non-significant negative values for both population groups. Notably, RNA L of the Chinese population was statistically significant at a P value < 0.01 based on Tajima’s D test and P value < 0.02 based on Fu and Li’s D and, F tests (Table 4). Whereas, negative values show polymorphisms within TSWV populations. It is also the indication of the selection purification and population expansion in all three genomic segments of TSWV.

**Table 4:** Statistical results from neutrality tests to evaluate demographic changes in the L, S and M segments of TSWV populations

TSWV Population	TSWV segment	Neutrality tests		
		Tajima’s *D*	Fu and Li’s *F*	Fu and Li’s *D*
**China**	L	-2.338*	-3.331**	-3.065**
	S	-1.734	-1.591	-1.241
	M	-1.804	-1.509	-1.060
**Global**	L	-0.900	-1.303	-1.222
	S	-0.551	-0.664	-0.607
	M	-0.649	-0.482	-0.358

*statistically significant at P < 0.01, ** statistically significant at P < 0.022.4.3

#### Estimation of gene flow and genetic differentiation

Nucleotide-based test statistics (Ks*, Kst*, Z* and Snn) were estimated to reveal the genetic differentiation of TSWV population (Table 5). All the tests were statistically non-significant at 0.01 < P < 0.05. Near zero value of Kst* (0.076, 0.255, and 0.215 for L, M, and S segments, respectively), depicted that there is no/ very low genetic difference (Table 5). Similarly, relatively lower values of the Z* test (5.236, 4.749, and 4.207 for L, M, and S segments, respectively) emphasized the same trend of less genetic differentiation (Table 4). Likewise, the values of Snn were also estimated near zero (0.932, 1.000, and 0.783 for L, M, and S segments, respectively) indicating that the population of TSWV is distinctly differentiated. Furthermore, Fst revealed the infrequent gene flow among the tested genomic segments by computing the near absolute values > 0.33 (0.334, 0.381, and 0.332 for L, M, and S segments, respectively). Whereas, Nm values < 1 highlighted the reduced gene flow, indicating increased genetic drift that results in regional population differentiation (Table 5).

**Table 5:** Estimates of gene flow and genetic differentiation for L, S, and M segments of TSWV populations originating from China and other countries

TSWV Segment	K_s_*	K_st_*	Z*	S_nn_	*F*_st_	Nm
L	4.584	0.076	5.236	0.932	0.334	0.50
S	63.403	0.215	4.207	0.783	0.332	0.50
M	101.82	0.255	4.749	1.000	0.381	0.41

Ks*, Kst*, Z*, and Snn: statistical tests that measure genetic differentiation, *Fst*: measures the extent of gene flow, Nm: successfully incoming migrants per generation

## Discussion

As a cash crop, tobacco has an impact on living for decades worldwide, where China ranks first as the largest producer having 50% of the global farmers [24]. The tobacco industry in China remains one of the most important sources of tax revenue for the central government [1]. Due to the huge impact of viral diseases on the final crop yield, especially TSWV resulting in heavy qualitative and quantitative losses, and the importance of tobacco as a cash crop, this study was focused to assess and identify the prevalence and population dynamics of TSWV in tobacco crops in China on a larger scale. For this purpose, full-length genome sequences of ten TSWV isolates from tobacco were analyzed from different regions including Hunan, Shandong, Shanxi, Sichuan, and Yunnan provinces; well known to have a major contribution to tobacco production in China [25].

Recently, the increasing trend of TSWV reports from China in different host plants has been observed, indicating its importance as an emerging interest to investigate the potential threat to agronomic crops as well as gardening and ornamental plants [26–28], but studies focusing the population dynamics and evolution of TSWV on large scale, especially in tobacco crops are relatively less, with one exception reporting full-length genome of a single TSWV isolate, infecting tobacco in Yunnan [5]. To fill this research gap, our study has investigated the genetic diversity and evolution of this important plant virus focusing on the main tobacco-growing regions of China including five provinces. Moreover, this is the first single study reporting ten full genome sequences of TSWV infecting a single host in China as well as around the globe. Also, six isolates were partially sequenced where five isolates contained full-length RNA L and M, with missing sequences of S segments (Table 11).

Owing to RNA segmented genome orientation, TSWV has great genomic diversity and a tendency to evolve making it one of the most devastating plant viruses by a great ability to evolve according to environmental stress [29]. TSWV is well established in many geographical areas of the world and therefore has extensive literature on molecular phylogenies that give the opportunity to better understand its evolutionary habits [30, 31]. Availability of a rapid and high number of sequencing data has presented the phylogenic analysis as a vital tool to study the distant relationships amongst species, the spread and origin of viral infection and migration configurations of viruses, through versatile statistical methods with their advantages and disadvantages [32]. In the current study, the maximum likelihood (ML) approach was implemented for the evolutionary analysis in the form of the bootstrap consensus phylogenetic tree formation. The tree depicted that newly reported Chinese isolates of TSWV were aligned separately from previously reported isolates, which revealed a clear tendency of newly reported TSWV isolates to occupy a nearly separate place in the phylogenetic trees of all three genomic RNAs i.e., L, M, and S (Figs. 2, 3 and 4), that pointed towards the divergent origin and distant relation among these and the rest of the world corresponding sequences. However, in case of RNA L and S, some newly reported Chinese isolates shared relatively close relationship with global isolates i.e., SCLS, YNHHMZ, YNKM-1. That indicates the possible migration of TSWV on a large scale and co-infection with its distantly related African and Korean isolates (Figs. 2 and 4).

In the case of RNA L, separate placement of three newly sequenced L isolates along with one previously reported Chinese isolate in clade A, having a close relationship with African isolate MG602671 showed that some reassortment events might take place between TSWV isolates from African and Chinese parental sequences. The idea has little support from the fact that these isolates were collected from different hosts i.e., tobacco, chrysanthemum, and tomato, also suggesting intensive interchange of inoculum or insect vector between these crop plants that might have related to the co-evolution of the virus. Whilst, all other sequences were divided into the Chinese population in clade B and the other GenBank retrieved isolates in clade A (Fig. 2). Moreover, ML tree of RNA M depicted the clear distinction between Chinese and world sequences by dividing them into two clades A, and B possessing TSWV from other countries and China, respectively. Likewise, the RNA S phylogeny showed that all the global sequences were clustered together in clade A, hosting one newly reported Chinese isolate YNHHMZ, sharing closeness with Spanish and American isolates. While, all the other Chinese isolates occupied place together in clade B, suggesting both recombination in general and reassortment in specific might have been involved during the inter-genomic shuffling and recombining of genomic material of S RNA, which often take place when the viruses overcome the resistance of host plant [33]. The evidence of involvement of non-structural (NSs) and nucleocapsid protein (NP) coding regions of RNA S of TSWV have been reported to break the resistance of *Tsw* gene in pepper [33, 34].

Furthermore, RNA L, M, and S showed nearly the same trend of phylogeny; an indication about the Chinese TSWV population possesses its own distinct behavior towards genome variability and evolutionary history that make it the best suited for the largest tobacco landscape in the world and make it different from rest of the world population. The trees also denoted that the evolutionary association and root of Chinese isolates were less divergent among themselves as compared to the world TSWV population, which might be an indication of excessive interchanging of genetic material among local isolates pertaining to recombination or reassortment during co-infection of single cells of distantly related TSWV isolates.

This consistency of newly identified isolates led to investigating the genetic diversity in-depth. Recombination analysis performed through recombination detection program (RDP; version 4) program calculated a total of seven recombination events, in the true sense referred to the reassortment in viruses with RNA segmented genomes [35, 36]. Both recombination and reassortment are also reported as evolution tools of TSWV [33, 37]. RNA L segment of European isolates depicted three recombination events having a relatively smaller recombinant area at the tail end. Interestingly, RNA M of the newly reported isolate had one recombination event but was highly significant and larger than L segment. This revealed that reassortment/ reshuffling of cell-to-cell movement protein (NSm) coding region potentially played important role in the evolution of YNKM-2 TSWV Chinese isolate, and acts as an indication of the future constraint of severe viral infection in tobacco crops with greater impact owing to genome diversity. The point to ponder is that the parental sequences (YNKMXD and YNHHKY) were also from newly identified isolates, revealing the onset of the local TSWV population to evolve vigorously overcoming the unfavorable conditions. Moreover, RNA S of three Italian sequences had the largest remnant area supporting the involvement of S segment in the evolution of TSWV through conferring great ability of resistance breaking [34, 38].

On contrary, the frequency of recombination/reassortment events in the evolution of RNA viruses is far less in nature [39]. Rather, it might involve a high number of vital mutations or large populations [35]. Additionally, it is inevitable for viruses with larger RNA-segmented genomes to replicate without fatal mutations [40, 41]. The debate is still searching for strong evidences, but it also cannot be excluded that reassortment is amongst the key players offering genome diversity and evolution of these viruses. The complete-genome reassortment analysis revealed that possible reassortment might have occurred at country level in L segment of YNKMXD isolate. While even less tendency was observed for Segment M, and S of newly reported TSWV isolates (Fig. 6).

The population dynamics analysis consisting of genetic diversity, demographic changes through neutrality tests, gene flow and genetic differentiation; further demonstrated that the Chinese population of TSWV is more conserved showing very less genetic variation. A statistical estimation of the genetic diversity of three genomic segments L, M, and S revealed that the L segment of the Chinese TSWV population was relatively more conserved, having a low number of Eta as compared to the global L segment. Whereas, the number of segregating sites was higher in case of M segment when compared with global population which might be an indication of the potential capacity of M RNA to mutate or evolve. The trend was also supported by RDP4 results, where M segment of newly reported Chinese isolate YNKM-2 depicted a highly significant recombination event detected by all seven algorithms (Fig. 5). Also, RAT confirmed the above findings for M segment of the same isolate (Suppl. Figure S2). While S RNA showed less divergence for both Chinese and global populations. Interestingly, the neutral evolution test statistics showed that all three tests (Tajima’s D, Fu and Li’s D and Fu and Li’s F) showed significant negative values for L segment of Chinese population, pertaining to the polymorphism and selection purification, especially in this region of TSWV genome.

Furthermore, gene flow and genetic differentiation test statistics revealed the same trend of low genetic differentiation among the TSWV population globally. The estimated values of nucleotide-based test statistics (Ks*, Kst*, Z* and Snn) depicted that there was no or very low genetic differentiation was observed among all three segments L, M, and S (Table 5). Whereas, Fst estimated the infrequent gene flow, supported by the Nm values < 1, a sign of reduced gene flow/ increased genetic drift.

## Conclusion

Conclusively, a maximum full-length genome count of TSWV isolated from a single host; tobacco, revealed that the Chinese population is more conserved among local isolates, i.e., building a versatile genome orientation to cope with different environmental conditions over a large cultivated landscape of tobacco in China. Genetic diversity and population dynamics analysis also revealed the conservation of TSWV population (more in case of Chinese population). Whereas, M segment was highlighted to be more capable of mutating or evolving as indicated by the results of RDP4, RAT, population dynamics, and phylogenetic analyses.

## Materials and methods

### Plant samples collection

**Fig. 7:**
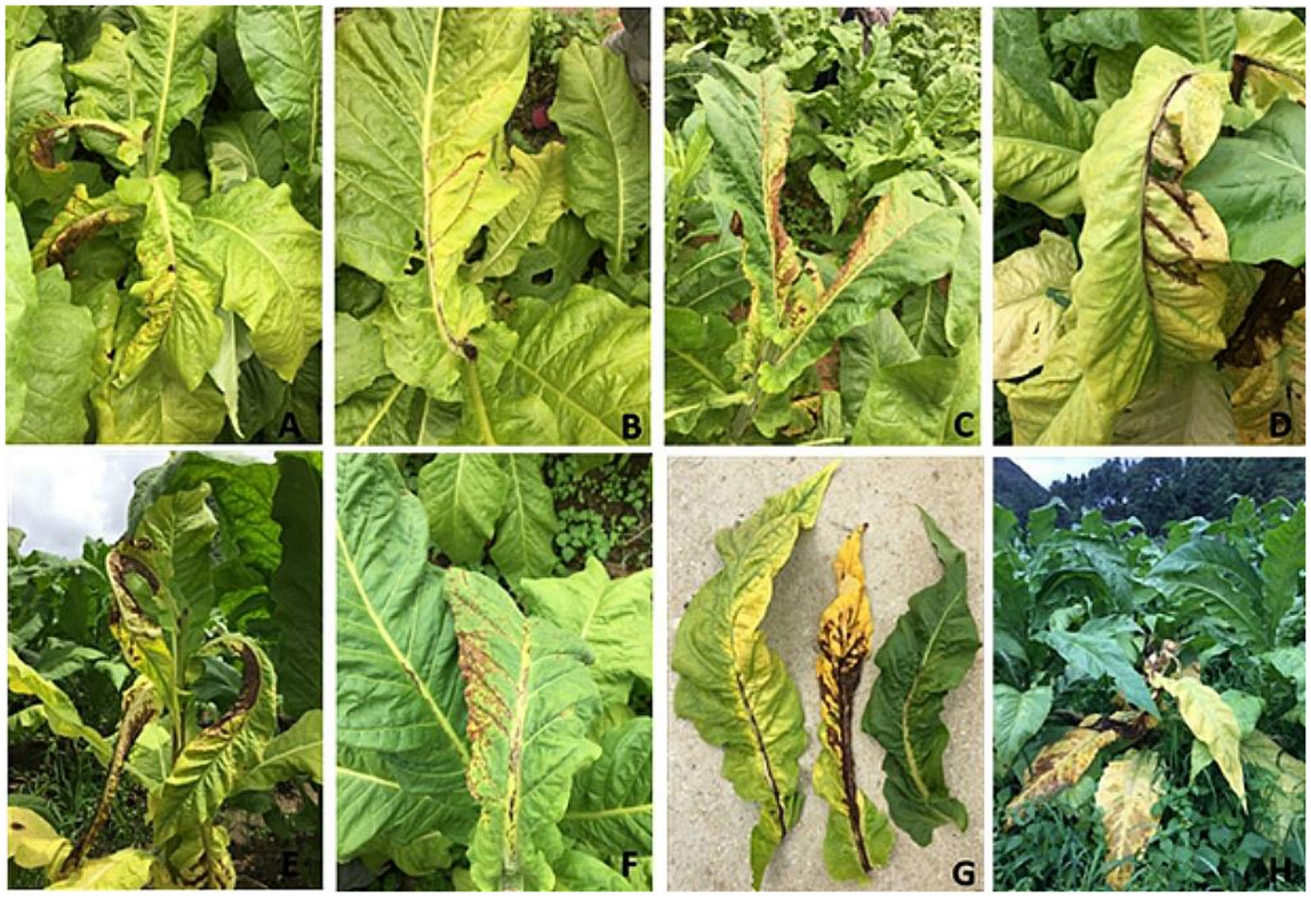
Orthotospovirus-like symptoms such as systemic necrosis resulting in the formation of a large area of dead tissues alongside the midrib, elongated necrotic spots on the midrib, leaf chlorosis, distorted apical buds, and leaf curling (A to H) were observed on tobacco plants during field survey

During the growing seasons of 2017–2020, a total of 214 tobacco leaf samples were collected from different locations in Gansu, Hunan, Shanxi, Shandong, Sichuan and Yunnan provinces. All the collected samples exhibited spotted wilt disease (SWD) symptoms (Fig. 7).

### Viral screening of plant samples

Total RNA was extracted from 156 selected tobacco leaf samples (representative of different visited locations during the three-year survey), through RNAiso Plus (Total RNA extraction reagent) (Takara) following the manufacturer’s protocol. For the detection of orthotospovirus, tobacco leaf samples were screened through a degenerate primer pair dTospo-F2 (5′-GATCAATCNAARTGGTCDGCWTC-3′) and dTospo-R2 (5′-CATDGCACAAGARTGRTAVACWGA-3′) to amplify an amplicon of 312-bp representing the part of the RdRp [42]. The samples that were positive for orthotospovirus with the above-mentioned generic reverse transcriptase- polymerase chain reaction (RT-PCR) assay, were subsequently tested for TSWV [5], impatiens necrotic spot orthotospovirus (INSV) [43] and tomato zonate spot orthotospovirus (TZSV) [44].

### PCR amplification of full-length TSWV genomic segments

For the TSWV-positive samples, a total of 17 primer pairs were constructed to obtain all three genomic segments i.e., L, M, and S of TSWV including nine primer pairs for L RNA, five for M RNA, and three for S genomic RNA (Table 1). For PCR-based full genome amplification of the TSWV sequence, cDNA was used as a template, and the primers mentioned in Table 6, were used to carry out separate PCR assays for the L, M, and S genomic segments, respectively. The PCR tube contained the following reagents: 2 × Taq Plus Master Mix (II) 20 µl, the upstream and downstream primers (1.6 µl each), cDNA 1.0 µl, and 17.4 µl ddH_2_O to make the total volume up to 40 µl. PCR cycling conditions were set as; pre-denaturation at 95 °C for 4 min; denaturation at 95 °C for 30 s, annealing for 30 s, extension at 72 °C for 1 min, number of cycles: 30 and final extension at 72 °C for 10 min. The amplified PCR products were analyzed by visualization in 1.5% agarose gel electrophoresis.

**Table 6:** Primers designed for full genome sequence analysis of TSWV

Serial No.	Primer name	Sequence (5′-3′)	Amplicon size (bp)	Tm (℃)^a^
1	TSWV-L-1 F	AGAGCAATCAGGTAACAAC	1257	51
	TSWV-L-1R	TTTTGGATTAAGTGGTCTTTC		
2	TSWV-L-2 F	GAAGATATTGAAAGAATAATTGATTC	1101	50
	TSWV-L-2R	ATTCAGGATTTTTAGCATAATTATTG		
3	TSWV-L-3 F	CAAGTATAGACATGTCTTCTCTGACTC	1286	55
	TSWV-L-3R	CCTAGATACTAATCAGATAATGCTTTC		
4	TSWV-L-4 F	TGAAGACATATACCCTAAGAAAGC	976	52
	TSWV-L-4R	CAGATAGGAAAGCCAATCTTG		
5	TSWV-L-5 F	CATCGGAAGCCATATCTATAAG	1158	54
	TSWV-L-5R	GCTCTCTGAATCTCATCTGTAGATA		
6	TSWV-L-6 F	CTCCTATAGAGCCGTTGTCTATATTAG	1161	55
	TSWV-L-6R	ATCATATAATTGCATGCTTTCACC		
7	TSWV-L-7 F	TTGGAACAGGTTTAATCATGG	1276	52
	TSWV-L-7R	TTCTCTGATATCATCATCTACAATCTC		
8	TSWV-L-8 F	GAATGACCACAGACAACAAAATG	1131	52
	TSWV-L-8R	CAATCAAGAACTTTATCAACTCACTTAG		
9	TSWV-L-9 F	GTGTTGAGGCTAGATGAGGAAG	916	55
	TSWV-L-9R	AGAGCAATCAGGTACAACTAAAAC		
10	TSWV-M-1 F	AGAGCAATCAGTGCATCAGA	955	55
	TSWV-M-1R	CTTCTTCTTCAACTGATCTCTCAAG		
11	TSWV-M-2 F	GCAAGCTGATAATTCCTAAAGG	1351	55
	TSWV-M-2R	AAGGAGATGACATGTCTTGGG		
12	TSWV-M-3 F	CCGCATAGAAGACAGCC	1276	55
	TSWV-M-3R	GTTATAGAAGGTCCTAATGATTGCA		
13	TSWV-M-4 F	GTTAACCCTAAAGAGCTTCCTG	979	52
	TSWV-M-4R	GAGAAGATCATGGGTTATTTGAT		
14	TSWV-M-5 F	CTTATCCAAGAAAAATTGATGC	1051	50
	TSWV-M-5R	AGAGCAATCAGTGCAAACAAAA		
15	TSWV-S-1 F	AGAGCAATTGTGTCATAATTTTATTC	1258	52
	TSWV-S-1R	TTGCAGATATCTTCACTGTAATTTAAG		
16	TSWV-S-2 F	CTCTGCTTGAAACTCACACATC	1097	52
	TSWV-S-2R	AAATGAATGAAGATCAGGTGAAG		
17	TSWV-S-3 F	YCCATAGCAATACTTCCTTTAGC	848	50
	TSWV-S-3R	AGAGCAATTGTGTCAATTTTATTC		

^a^ annealing temperature of the primers

### PCR product recovery, sequencing and data analysis

The targeted fragments were purified from 1.5% agarose gel according to the protocol of QIAquick Gel Extraction Kit (Qiagen). The recovered amplicons of interest were then ligated to pMD18-T vector followed by transformation into *E. coli* strain DH5α. The positive clones were screened using a medium containing ampicillin antibiotic. After the colony PCR was verified, the bacterial solution was sent to the company for sequencing. The obtained sequences were then subjected to bioinformatics analysis. MegAlign software available in DNAStar package (DNASTAR, Madison, USA) was used to analyze the sequences. BioEdit (Version 7.2) was then used to edit the obtained sequence alignments (http://www.mbio.ncsu.edu/bioedit/bioedit.html). The cleaned sequences of three genomic RNAs (L, M, and S) were then deposited in GenBank database. Amino acid (aa) sequence identity for L, M, and S RNA was calculated using Geneious Prime 2021.2 (Biomatters Ltd., Auckland, New Zealand) (Suppl. table S1-S3). For phylogenetic and recombination analysis, newly identified and previously reported nucleotide sequences of all three newly identified RNA segments along with the selected corresponding GenBank sequences of TSWV were aligned through ClustalW [45] provided in MegaX software [46].

### Molecular phylogenetic analysis

Mega format files containing full-length nucleotide sequences of 37 RNA L, 30 RNA M, and 24 RNA S were run separately in MegaX software [46] to perform phylogenetic analysis between Chinese isolates reported herein and other TSWV corresponding sequences in GenBank. Maximum likelihood (ML) trees were constructed using the Bootstrap method (with 1000 replicates) to assess the statistical significance as well as the reliability of each node in the phylogenetic tree.

### Recombination analysis

Potential recombination events were predicted in all newly reported full-length genomic segments i.e., L, M, and S of TSWV together with corresponding GenBank sequences, using the RDP4.101 software [47]. The recombination/reassortment events, potential parental sequences of recombinants, and breakpoints were analyzed using seven different detection algorithms i.e., Bootscan, Chimaera, Geneconv, maximum Chi-square Rdp, SisterScan, and 3Seq, implemented in RDP4 package. The recombination events were considered reliable if detected by at least four different methods. The results having P-values < 0.05 were considered significant. Recombination Analysis Tool (RAT) was used to rectify the findings of RDP4 findings [48].

### Genetic diversity and population dynamics of TSWV isolates

#### Estimation of genetic diversity

The genetic diversity of all three genomic segments L, M, and S of TSWV was analyzed based on geographical location using DNASP6. The analysis was performed by computing the number of segregating sites, haplotype number, haplotype diversity, average number of nt differences, average nt diversity, the total number of mutations, and Watterson’s theta. The analysis consisted of both the Chinese (including both novel and previous GenBank sequences) and the global sequences, assigning two groups of population.

#### Statistical evaluation of demographic changes through neutrality tests

The number of polymorphic (segregating) sites, total number of mutations, nucleotide diversity, and Watterson’s theta (per sequence and per site) were used for validating the neutrality hypothesis using different statistical tests i.e., Tajima’s D, Fu, and Li’s D and Fu, and Li’s F between both population groups. Tajima’s D test considers the differences between, Tajima’s estimator and Watterson’s estimator [49]. The test shows positive values for an abundance of segregating alleles, while negative values indicate rare alleles. Fu and Li’s D test analyze the total mutation sites and singleton mutation sites while Fu and Li’s F test compute the difference in singleton mutation sites and the average number of nucleotide differences [50]. The resulting negative values of these tests indicate low-frequency segregation [51].

#### Estimation of gene flow and genetic differentiation

To compute the population differentiation the most powerful considered statistical tests i.e. Ks*, Kst*, Z*, and Snn were used. These tests are highly reliable when the difference among the mutating population in small sample sizes is in question. Where, The Ks* indicates the average number of differences between nt sequences remaining unbiased of geographical origin, while the near zero value of Kst* under the null hypothesis, shows that there is no genetic difference and vice versa. Whereas, lower values of Z* test indicate less genetic differentiation and higher values are the measurements of higher genetic differences. On the same note, the values of Snn (nearest neighbor statistical test) near one, indicate the distinctly differentiated populations. The fixation index (Fst) was used to compute the extent of gene flow and the number of migrants incoming successfully per generation (Nm). The values of these statistics are calculated between 0 to1, where 0 indicates no genetic diversification and vice versa. The absolute value > 0.33 shows infrequent gene flow between the tested populations. Whereas, Nm values < 1 highlight the reduced gene flow indicating increased genetic drift that results in regional population differentiation. Additionally, Fst values < 0.25 were not observed to conclude the frequent gene flow/ no genetic differentiation [49, 51].

#### Source of viral inoculum

Samples were collected from N. tabacum plants showing characteristic symptoms of TSWV from Changsha and Kunming localities in Yunnan province. TSWV infection was confirmed through RT-PCR assay using primer pair NF302:5’-GGTCAGGCTTGTTGAGGAAAC-3’ and NR575: 5’ TTCCCTAAGGCTTCCCTGGTG-3’ yielding an amplicon of 350 bp in size. The selected samples were further used for mechanical inoculation, first in N. tabacum followed by tomato plants (Solanum lycopersicum) (primary host plant). All the experiments were performed in a growth chamber with controlled temperature and relative humidity conditions. The inoculated plants were regularly observed for the development of symptoms. TSWV infection was confirmed through RT-PCR assay.

#### Raising nursery of test plants

Seedlings of tomato and tobacco plants were raised in the greenhouse for the mechanical inoculation experiment. All the seeds were kindly provided by Plant Production Department, Tobacco Research Institute (TRI). Eight to ten seeds were sown in each plastic pot containing the mixture of peat moss and sterilized soil (ratio; 80:20). The experiment was carried out in a growth chamber with controlled growing conditions. Seedlings having uniform growth were transferred to new pots.

#### Preparation of TSWV inoculum

Freshly infected leaves from tobacco plants were pre-frosted in liquid nitrogen and ground [tissue: buffer; 1:6 (weight/ volume)] with cold and freshly prepared potassium phosphate buffer (0.01 M), having pH of 7.0, consisting of 0.01 M 2-mercaptoethanol and 0.2% sodium sulfite, in a pre-chilled pestle and mortar. The ground inoculum was filtered through a muslin cloth to remove the debris. Celite 545 (Fisher Scientific) and Carborundum (Fisher Scientific) with a ratio of 1:2. The prepared inoculum was placed on ice till its use for inoculation of the test plants [52].

#### Mechanical inoculation

The inoculum was gently rubbed using cotton swabs on the leaf surface and rinsed with tap water to remove the leftover salt. The plants were kept in a growth chamber and observed for symptoms development. Samples were then collected after four weeks from symptomatic and asymptomatic plants to test the TSWV infection using primer pair (TSWV-NF / R).

### Electronic supplementary material

Below is the link to the electronic supplementary material.

**Supplementary Material 1: Figure S1.** Representative 1.5% agarose gel showing expected amplicons of all three genomic RNAs of YNHH isolate of TSWV. **Figure S2.** Recombination Analysis Tool (RAT) output for L segment of TSWV: (A) KP008132 (pepper-Spain), (B) MK348942 (pepper-Italy). **Figure S3.** Recombination Analysis Tool (RAT) output for M segment of newly reported YNKM-2 isolate of TSWV. **Figure S4.** Recombination Analysis Tool (RAT) output for S segment of TSWV: (A) MG989674 (pepper-Italy), (B) MG989675 (pepper-Italy). **Figure S5.** Graphical representation of potential reassortment breakpoints detected among mixed TSWV genomic segments: L, M, and S RNAs

**Supplementary Material 2: Table S1.** Amino acid identity percentage of RNA L

**Supplementary Material 3: Table S2.** Amino acid identity percentage of RNA M

**Supplementary Material 4: Table S3.** Amino acid identity percentage of RNA S

## Data Availability

Full-length sequences of all newly constructed genomic segments of TSWV isolates were submitted to GenBank with the accession numbers mentioned in the main text by visiting https://www.ncbi.nlm.nih.gov/nuccore/.

## Abbreviations

FAOSTAT: Statistics Division of Food and Agriculture Organization of the United Nations
TSWV: tomato spotted wilt orthotospovirus
L: large genomic RNA
M: medium genomic RNA
S: small genomic RNA
RDP: recombination detection program
RdRp: RNA dependent RNA polymerase
NSm: Cell-to-cell movement protein
GP: glycoprotein precursor
NSs: non-structural protein
N: nucleocapsid protein
RNPs: ribonucleic capsid proteins
eEF1A: eukaryotic elongation factor 1 A
SWD: spotted wilt disease
RT-PCR: reverse transcriptase- polymerase chain reaction
INSV: impatiens necrotic spot orthotospovirus
TZSV: tomato zonate spot orthotospovirus
ML: Maximum likelihood
TRI: Tobacco Research Institute
aa: amino acid
RAT: recombination analysis tool
nt: nucleotide
NJ: neighbor-joining
