# Supplementary material for: Insights into the genetic variability and evolutionary dynamics of tomato spotted wilt orthotospovirus in China
Source: BMC Genomics. 2024 Jan 8;25:40. doi: 10.1186/s12864-023-09951-9 (PMC10773106; doi:10.1186/s12864-023-09951-9)

**
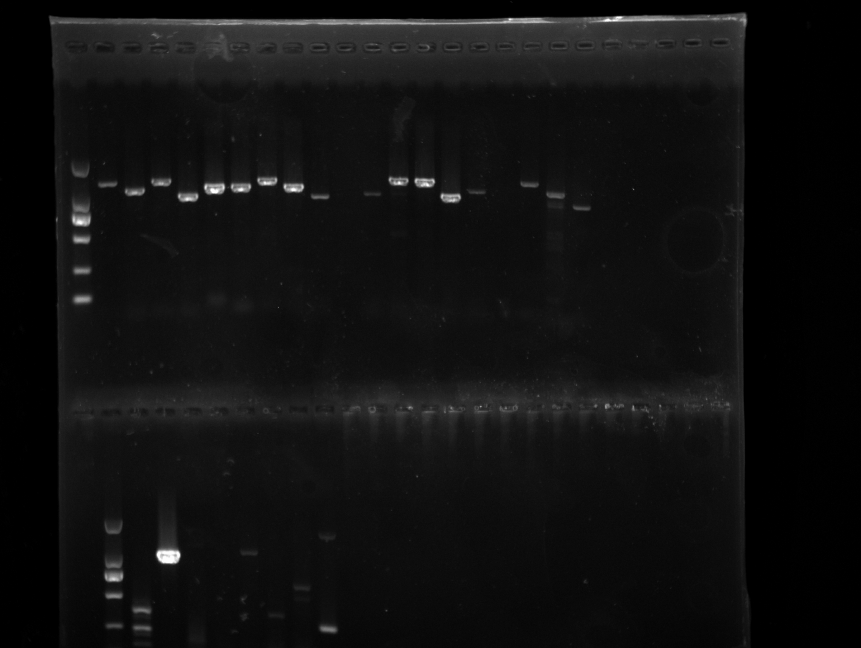
**

**Figure S1.** Representative 1.5% agarose gel showing expected amplicons of all three genomic RNAs of YNHH isolate of TSWV

**
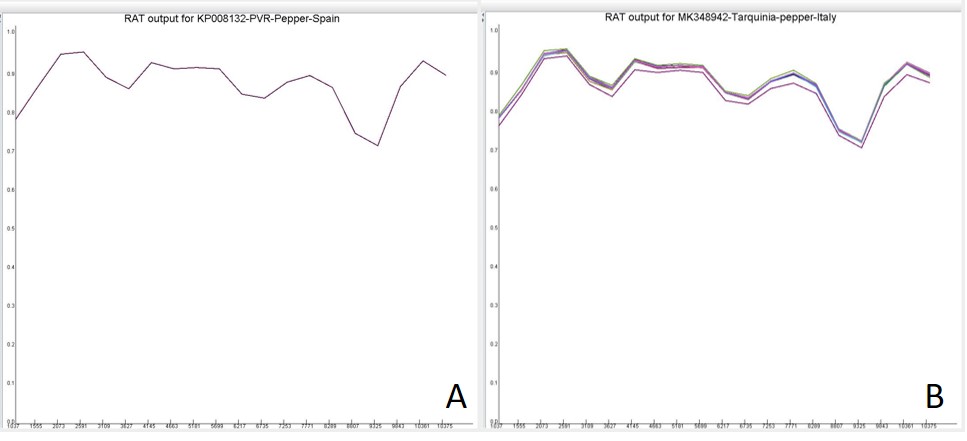
**

**Figure S2.** Recombination Analysis Tool (RAT) output for L segment of TSWV: (A) KP008132 (pepper-Spain), (B) MK348942 (pepper-Italy)


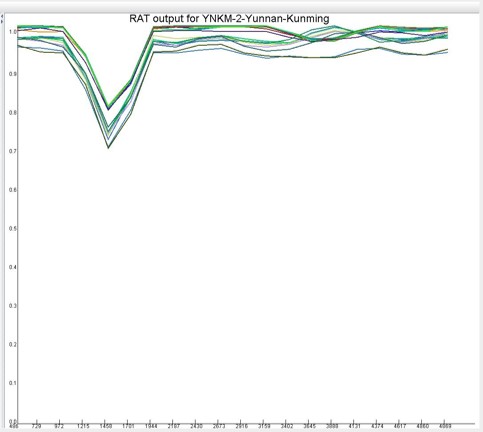


**Figure S3.** Recombination Analysis Tool (RAT) output for M segment of newly reported YNKM-2 isolate of TSWV

**
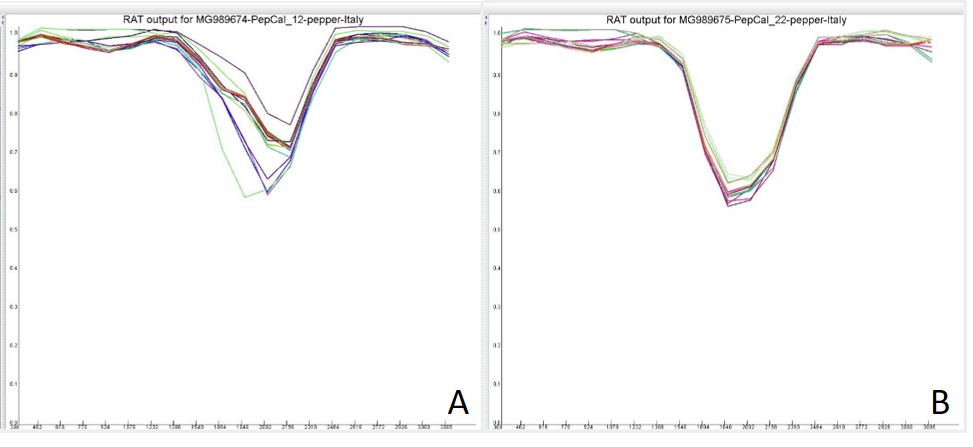
**

**Figure S4.** Recombination Analysis Tool (RAT) output for S segment of TSWV: (A) MG989674 (pepper-Italy), (B) MG989675 (pepper-Italy)

**Figure S5.** Graphical representation of potential reassortment breakpoints detected among mixed TSWV genomic segments: L, M, and S RNAs
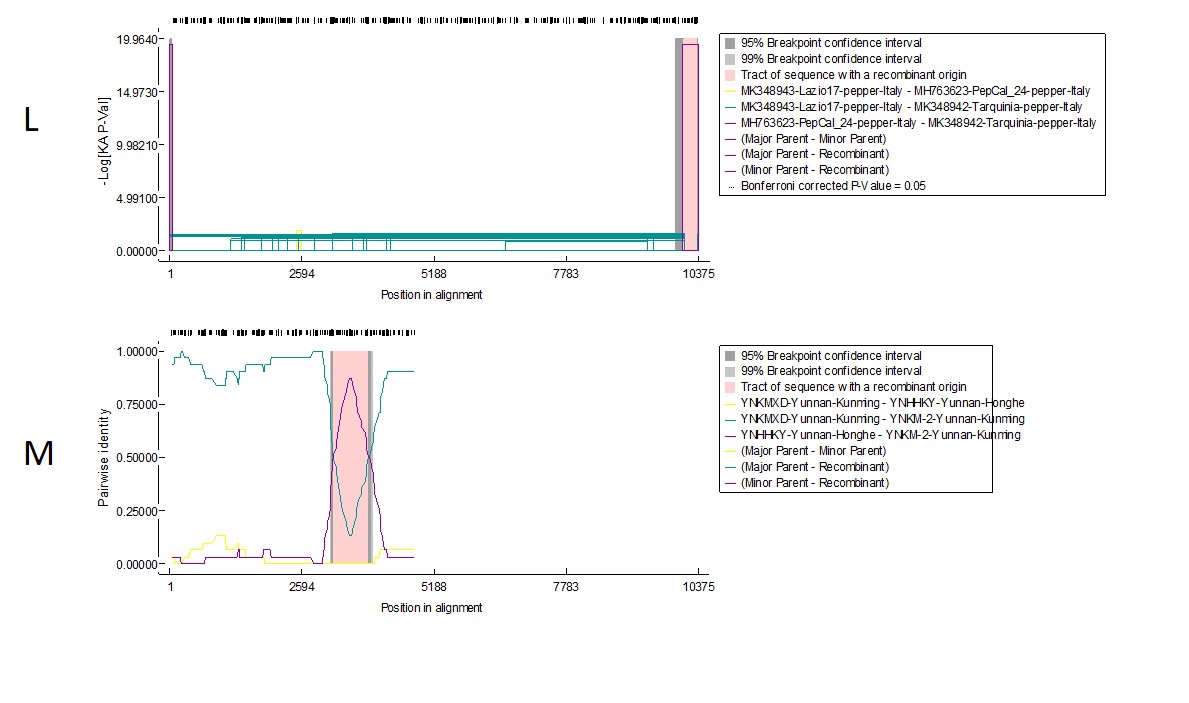

Supplement: Supplementary file 1 — Supplementary Material 1: Figure S1. Representative 1.5% agarose gel showing expected amplicons of all three genomic RNAs of YNHH isolate of TSWV. Figure S2. Recombination Analysis Tool (RAT) output for L segment of TSWV: (A) KP008132 (pepper-Spain), (B) MK348942 (pepper-Italy). Figure S3. Recombination Analysis Tool (RAT) output for M segment of newly reported YNKM-2 isolate of TSWV. Figure S4. Recombination Analysis Tool (RAT) output for S segment of TSWV: (A) MG989674 (pepper-Italy), (B) MG989675 (pepper-Italy). Figure S5. Graphical representation of potential reassortment breakpoints detected among mixed TSWV genomic segments: L, M, and S RNAs [file 12864_2023_9951_MOESM1_ESM.docx]
